# Mechanisms of oral ciprofloxacin-induced depressive-like behavior and the potential benefit of lactulose: A correlation analysis

**DOI:** 10.1016/j.toxrep.2025.101920

**Published:** 2025-01-20

**Authors:** Havizur Rahman, Kusnandar Anggadiredja, Lucy Sasongko

**Affiliations:** a Department of Pharmaceutics, School of Pharmacy, Institut Teknologi Bandung, Bandung 41116, Indonesia; b Department of Pharmacology and Clinical Pharmacy, School of Pharmacy, Institut Teknologi Bandung, Bandung 41116, Indonesia; c Department of Pharmacy, Faculty of Medicine and Health Sciences, University of Jambi, Jambi 36361, Indonesia

**Keywords:** Mechanisms, Analytical, Methods, Oral ciprofloxacin, Behavior, Depression

## Abstract

Prolonged administration of antibiotics may be associated with depression due to the potential risk of dysbiosis. Thus, the restoration of microbial balance, through administration of prebiotics, might overcome the problem. This study investigated the mechanisms of antibiotic-induced depression, which were explored through statistical correlation analysis. The potential benefit of lactulose, a prebiotic, on this behavioral disorder was further assessed. The rats were assigned to groups receiving 102.8 mg/kg ciprofloxacin daily for 1, 8, 15, or 22 days. A different group of rat was given the same regimen for 8 days accompanied with lactulose at 2056 mg/kg. Upon completion of ciprofloxacin administration, the rats were tested for depression-like behavior (forced swimming test, FST; and sucrose preference test, SPT). They were then sacrificed for biochemical assessment in the hippocampus and prefrontal cortex. The mechanism studies revealed significant correlation between SPT vs. serotonin in the hippocampus, and SPT vs. serotonin, cortisol, NF-κB in the prefrontal cortex. Meanwhile, FST was significantly correlated with serotonin in the hippocampus and the prefrontal cortex, while in the prefrontal cortex it was significantly correlated with cortisol, NF-κB, and IL-6. Based on the afore-mentioned results, it was found that lactulose improved FST by targeting serotonin in the hippocampus. This study indicate that ciprofloxacin induce depression-like behavior via modulation of several neurotransmitter system as well as proinflammatory cytokines in the hippocampus and prefrontal cortex. The results further suggest the potential of lactulose to improve this behavior.

## Highlights

• Oral ciprofloxacin causes depressive like behavior.
• Serotonin, cortisol, IL-6, and NF-kB in the brain affect depression-like behavior.
• Lactulose, prevents depression-like behavior by modulating serotonin in hippocampus.

## Graphical Abstract

**Figure:**
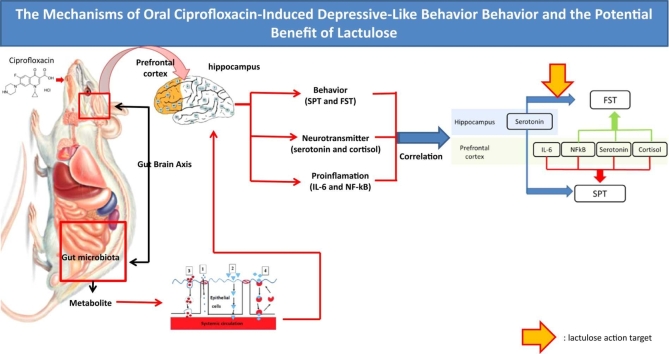

## 1 Introduction

The relationship between the gut microbiota and the brain, known as the gut-brain axis [1], has emerged as an exciting area of research. Recent studies have shown that antibiotic use can disrupt the balance of the gut microbiota, potentially leading to a variety of neurological disorders [2], [3], [4], [5], [6]. Of particular interest is the association between antibiotic administration and depressive-like behavior, a condition characterized by a spectrum of symptoms, including sadness, anhedonia, fatigue, and even suicidal thoughts, which often coincide with disturbances in the abundance of the gut microbiota [7].

Ciprofloxacin is one of the most commonly prescribed oral antibiotics. Depending on the indication, its duration of use as an anti-infective ranges from a few days to several weeks [8], [9]. Due to its long-term use and broad-spectrum antibiotic properties, there is concern about the potential for ciprofloxacin to disrupt the gut microbiota, which can lead to dysbiosis and microbial imbalance [10], [11]. This antibiotic affects the brain by producing proinflammatory metabolites produced by the disturbed gut microbiota [12], [13], [14].

There are concerns about inadequate monitoring of long-term ciprofloxacin use, particularly among outpatients receiving oral formulations [15]. In addition, limited awareness of depressive behavior and diagnostic inaccuracies further exacerbate the risk of therapeutic errors, especially considering cultural and regulatory barriers for healthcare workers [16], [17], [18], [19].

*Lactulose* is a disaccharide shown to remain unhydrolyzed by intestinal enzymes and acts as a prebiotic for beneficial Bifidobacterium species in the colon [20], [21]. Its efficacy in relieving constipation and promoting a healthy microbial balance has led to the hypothesis that lactulose may prevent dysbiosis and depressive-like behavior in patients on long-term oral ciprofloxacin therapy [22], [23].

This study aimed to elucidate the mechanism of depressive-like behavior induced by oral ciprofloxacin administration, explored through statistical correlation analysis, which we consider a novel approach. We further extend the study on the target of ameliorating action of lactulose on depressive-like behavior in a rat model.

## 2 Experimental procedures

### 2.1 Drugs and test substances

Ciprofloxacin Hydrochloride was obtained from Sanbe Farma (Bandung, Indonesia), and lactulose was obtained as the commercial product Lactulax (Ikapharmindo Putramas, Indonesia).

### 2.2 Animals

Adult male Wistar rats (weighing 200–250 g) were purchased from the School of Life Science and Technology, Institut Teknologi Bandung (ITB). Rats were kept in an animal laboratory at the School of Pharmacy (ITB) at 23 ± 2 °C, 40–60 % humidity, with 12:12 h dark and light cycles; food and drink were available *ad libitum*. The use and care of the animals were approved by the Ethics Committee on the Use of Experimental Animals of the Institut Teknologi Bandung (KEP/I/2022/IX/H140922HR/TDPS)

### 2.3 Experimental design

Following a one-week acclimatization period, the rats were randomly divided into five groups, with four rats per group. Groups H1 (S), H8 (S), H15 (S) and H22 (S) were administered ciprofloxacin for 1, 8, 15 and 22 days, respectively. Group H8 (S+Lac) was given ciprofloxacin for eight days concurrently with lactulose. The dose of ciprofloxacin was 102.8 mg/kg BW/day, while lactulose was 2056 mg/kg BW/day, both administered orally. The administration dose of ciprofloxacin and lactulose is based on the dose commonly used in humans [20], [24], [25], and the dose has been converted into the applicable dose for rats to ensure the safety of the administered dose [26]. Ciprofloxacin hydrochloride solution was prepared by dissolving the active pharmaceutical ingredient (API) in distilled water.

**Fig. 1:**
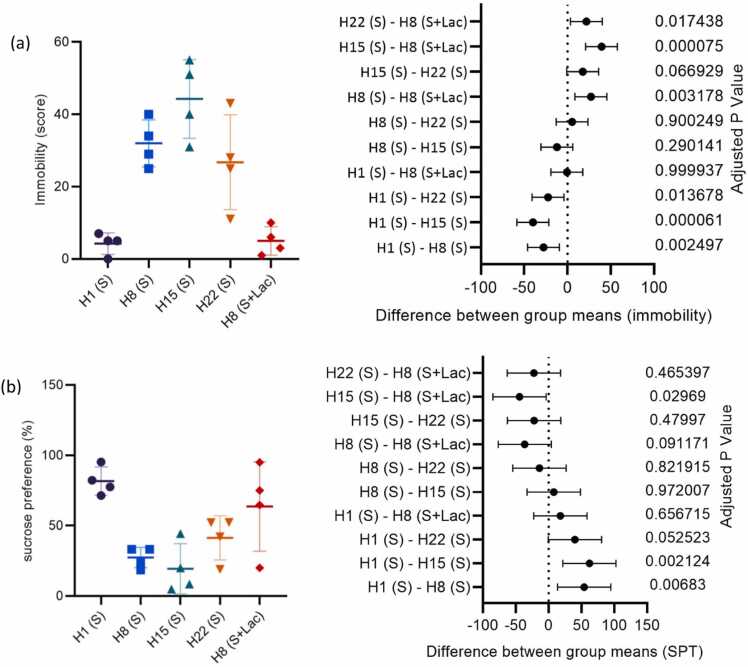
Effect of duration of ciprofloxacin administration, and lactulose on depression-like behavior (a) immobility in FST experiment, and (b) sucrose preference test.

The duration of oral ciprofloxacin induction time was selected based on the findings of several articles that utilized ciprofloxacin as an inducer of depression-like behavior. The articles indicated that 7, 14, and 21 days were optimal induction times [27], [28], [29]. From the pattern of depression-like behavior obtained (Fig. 1a), the duration of onset of depressive-like behavior was determined, which was day 8 of ciprofloxacin administration. Then, this was used as the basis for the duration of lactulose administration as a depression-like behavior-preventing agent. Furthermore, the administration of ciprofloxacin for eight days was employed as a reference point for determining the duration of lactulose administration, given that the maximum duration of lactulose administration commonly prescribed for humans is one week in the absence of supervision. Consequently, the administration of ciprofloxacin for 15 days cannot be used as a reference for the duration of lactulose administration, despite the observation that ciprofloxacin administration for 15 days exhibited higher depressive behavior than that observed with eight days. The dose of lactulose utilized in this study is commonly used in administering laxatives due to the enhanced assurance of the dose's safety.

### 2.4 Behavior test

#### 2.4.1 Forced-swim test

The swim test was performed 50 min following the final dose of oral ciprofloxacin. Each rat was placed for 5 min in an aquarium (40 x 40 x 40 cm) having a water depth of 30 cm, at 23–25 °C. The water in the aquarium was freshly replaced prior to each test. The swim test was carried out for 5 min (previously, a pretest was conducted 3 days before the actual test for 5 minutes). During the test, immobility (movement required only to keep the head above the water) was recorded using a stopwatch. After the training and test sessions, the animals were dried using towels and placed in heated cages [30]. Scoring is conducted at five-second intervals, with the dominant behavior serving as the unit of measurement [31].

#### 2.4.2 Sucrose preference test

Following the swim test, the sucrose preference test (SPT) was conducted. The SPT pretest was carried out three days prior to the actual test, which was conducted over 24 h to prevent neophobia. In the actual test, two bottles containing 0.75 % sucrose solution and distilled water, respectively, were weighed and provided to each rat for four hours. The total volume of sucrose solution consumed was determined and used as a parameter of hedonic behavior [32].

### 2.5 Quantification of neurotransmitters and proinflammatory cytokines

Fifty min after completion of the sucrose test, the rats were sacrificed in a CO_2_ chamber. The brain was collected, and the hippocampus (hippo) and prefrontal cortex (pre fc) were extracted. Preparation was carried out until a tissue homogenate was obtained. Tissues were rinsed in ice-cold PBS (pH 7.4), minced and homogenized in phosphate- buffered saline (the ratio of tissue (g) to PBS (ml) = 1:9). The tissues were kept at 4 °C for 10 min and then at room temperature for 5 min. The homogenates were then centrifuged for 5 min at 5000 rpm, and the supernatant was stored at −80 °C until analysis. The levels of serotonin, cortisol, IL-6 (Interleukin-6), and NF-κB (nuclear factor kappa light chain enhancer of activated B cell) in the tissue homogenate were measured using an ELISA kit (BT-Lab, Shanghai, China).

### 2.6 Statistical analyses

The levels of neurotransmitters and proinflammatory cytokines were compared using a one-way ANOVA test, followed by a Tukey test. A normality test was previously conducted using the Shapiro-Wilk test, which indicated that all data were normally distributed. The correlation between proinflammatory cytokines, neurotransmitters, and depression-like behavior was determined using the Pearson correlation test. Following the completion of correlation tests on all variables, the variables that significantly affect the forced swim test (FST) and the sucrose preference test (SPT) were identified. These variables also significantly affect the mechanism of action of the inducer in triggering depression-like behavior. To ascertain the target action of lactulose, a correlation test was conducted on lactulose from all variables that play a role in the mechanism of action that induces depressive behavior. The percentage difference between ciprofloxacin administration for 8 days, with and without lactulose, was employed to determine the activity of lactulose in inhibiting depressive behavior. A p-value of less than 0.05 was deemed statistically significant.

## 3 Results

### 3.1 Effect of duration of ciprofloxacin administration on depression-like behavior and potential preventive action of lactulose

Fig. 1a shows that the level of immobility increased with increasing ciprofloxacin induction time up to 15 days of administration, followed by a decrease at 22 days. Immobility was significantly more extended (p < 0.05) in groups receiving ciprofloxacin administration for 8 and 15 days compared to the 1 day group. The mean difference between immobility scores on day 1 and day 8 of ciprofloxacin administration was 15.38 (95 % CI: −46.19 to −9.306; p = 0.002497). Meanwhile, the difference between days 1 and 15 of ciprofloxacin administration was 35.9 (95 % CI: −58.44 to −21.56; p = 0.000061).

The immobility observed in the ciprofloxacin administration group for one day yielded an average value of 35 s. A review of the literature on healthy rats indicates that immobility occurs in the range of 28–97 s [33], [34], [35]. This suggests that the administration of ciprofloxacin for one day produced the same depression-like behavior as healthy conditions.

The sucrose preference test (Fig. 1b), in which there was a significant decrease in preference score (p < 0.05) in groups receiving ciprofloxacin and a reversion to normal in the score with H8 (S+Lac) group. A review of the literature on sucrose preference in healthy rats reveals a range of percentages, with values between 73 % and 85 % [33], [34], [36]. This indicates that the H1 (S) group exhibits the same depression-like behavior as that observed in healthy conditions.

The results of the behavioral tests, which employed the force swimming test (immobility), indicated that the administration of lactulose resulted in a statistically significant difference (p < 0.05) when compared to H8 (S) group, with a decrease in immobility of 84.38 % (Fig. 1a). However, the results differed when the sucrose preference test was employed (Fig. 1b), which demonstrated no significant difference (p > 0.05). Nevertheless, the SPT of the H8 (S+Lac) group, in comparison with the H8 (S) group, exhibited a reduction of 56.93 %. Furthermore, the H8 (S+Lac) group demonstrated SPT results that were not statistically different from the H1 (S) group, with a mean difference of 17.96 % (95 % CI: −22.64–58.56; p = 0.656715). This has demonstrated the efficacy of lactulose in preventing depression-like behavior. Nevertheless, in light of the preliminary nature of these findings, further research with a larger sample size is required to substantiate the observed effectiveness.

### 3.2 Effect of duration of ciprofloxacin administration and lactulose on proinflammatory cytokine levels

**Fig. 2:**
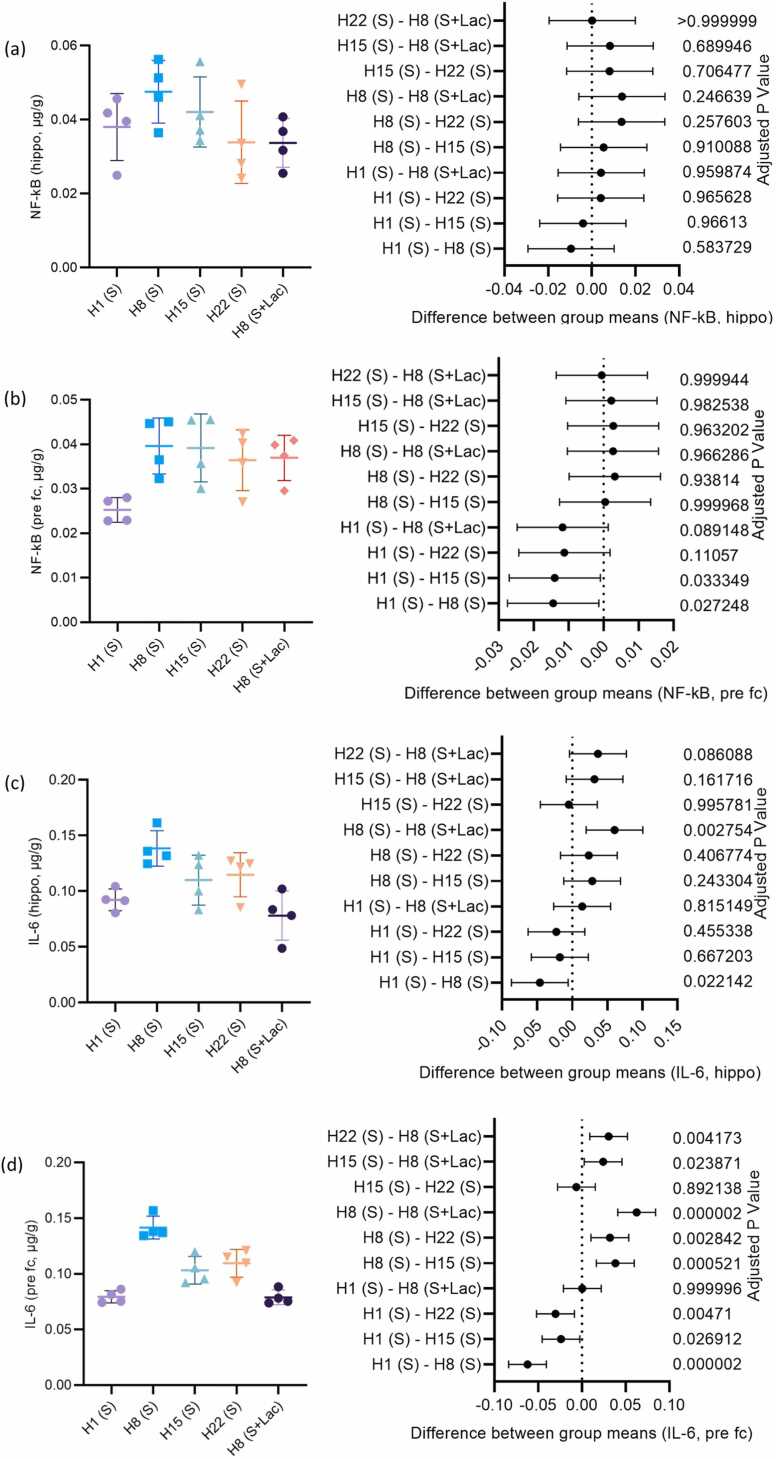
Effect of duration of ciprofloxacin administration, and lactulose on proinflammatory cytokine levels in the hippocampus (a, c) and prefrontal cortex (b, d).

As shown in Fig. 2a, hippocampus NF-κB levels following ciprofloxacin administration for 8, 15, and 22 days were higher, though not statistically significant, than that after 1 day. However, prefrontal cortex levels (Fig. 2b) were significantly increased in the 8 and 15 day-induced groups compared to the 1-day group. The average ratio of prefrontal cortex NF-κB levels for day 1 compared to day 8 of ciprofloxacin administration was 0.6369 (95 % CI: −0.02744 to −0.001357; p = 0.022142). Meanwhile, the average ratio for day 1 compared to day 15 of ciprofloxacin administration was 0.6442 (95 % CI: −0.02723 to −0.0006692; p = 0. 0.033349).

Hippocampus IL-6 levels in the group receiving 8-day treatment were significantly higher than those of the one day-treated group (Fig. 2c). The average ratio for day 8 compared to day 1 of ciprofloxacin administration was 1.5 (95 % CI: −0.08675 to −0.005642; p = 0.022142). Meanwhile, the average ratio for 8 days of ciprofloxacin compared to 8 days of concomitant lactulose administration was 1.77 (95 % CI: 0.01979–0.1009; p = 0.002754).

As presented in Fig. 2d, a similar pattern of changes in IL-6 levels was observed in the prefrontal cortex, except that the levels in groups receiving 15-day and 22-day ciprofloxacin administration were significantly lower than those of the eight day-induced group. The average ratio for 15-day compared to 8-day ciprofloxacin administration was 0.73 (95 % CI: 0.01673–0.05994; p = 0.000521). Meanwhile, the average ratio for day 15 compared to day 22 of ciprofloxacin administration was 0.958 (95 % CI: −0.02791–0.01530; p = 0.892138).

Hu et al. [37] reported IL-6 levels in the hippocampus of healthy rats to be approximately 10 pg/mg tissue. The research demonstrated that IL-6 levels in the hippocampus of the brain following the administration of ciprofloxacin for one day were 0.1 microgram/gram tissue, which is equivalent to 100 pg/mg tissue. The present results demonstrated that the administration of ciprofloxacin for a single day caused elevated IL-6 levels in the hippocampus relative to healthy conditions.

### 3.3 Effect of duration of ciprofloxacin administration and lactulose on neurotransmitter levels

**Fig. 3:**
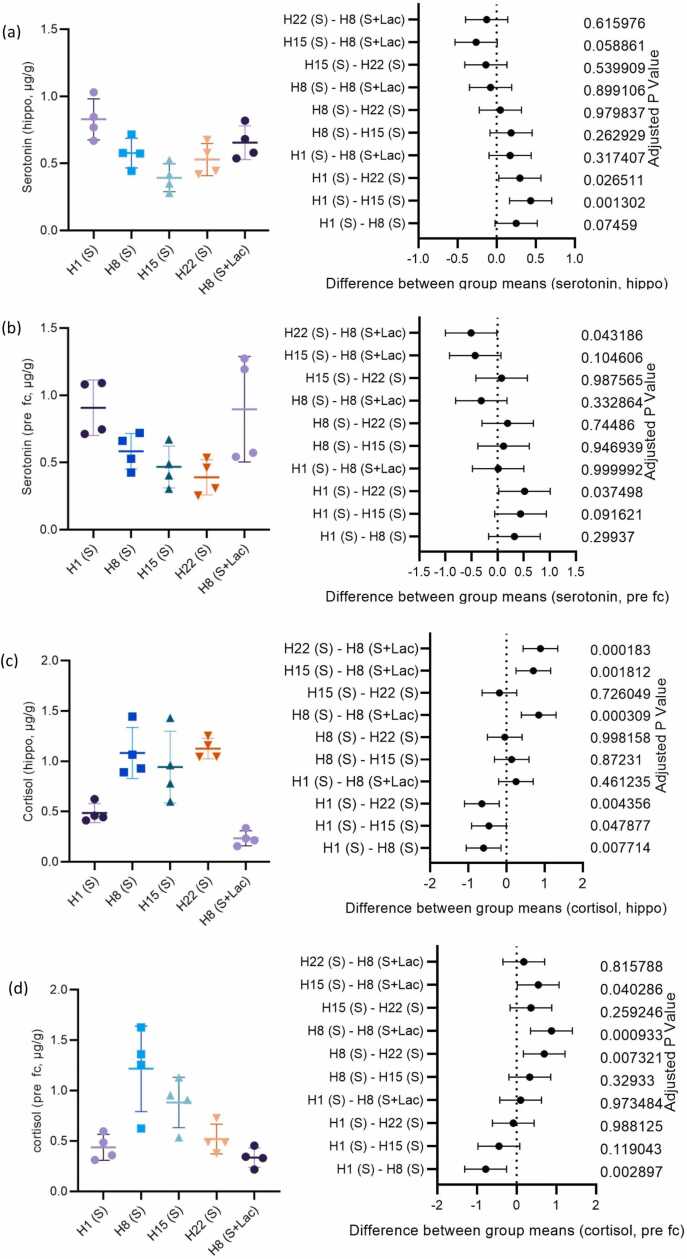
Effect of duration of ciprofloxacin administration, and lactulose on neurotransmitter levels in the hippocampus (a, c) and prefrontal cortex (b, d).

As Fig. 3a shows, hippocampus serotonin concentration decreased in the groups receiving ciprofloxacin, with the lowest in the H15 (S) group. The H8 (S+Lac) group tended to revert the concentration to the H1 (S) group. Analysis of the prefrontal cortex demonstrated a similar pattern, but the lowest level was found in the H22 (S) group, as presented in Fig. 3b. Measurement of cortisol levels in both the hippocampus and the prefrontal cortex showed increased hormone levels with more extended ciprofloxacin treatment, and that coadministration with lactulose had no effect (Figs. 3c and 3d).

Tu el al. [38] reported that serotonin levels in the hippocampus of healthy rats ranged from 2.52 micromole/gram tissue. The research conducted demonstrated that the serotonin level in the hippocampus of the brain following the administration of ciprofloxacin for one day was 0.83 microgram/gram tissue. The present results demonstrated that the administration of ciprofloxacin for a single day resulted in decreased serotonin levels in the hippocampus compared to healthy conditions.

### 3.4 Correlation analysis of proinflammatory cytokine and neurotransmitter levels with depression-like behavior induced by ciprofloxacin

**Fig. 4:**
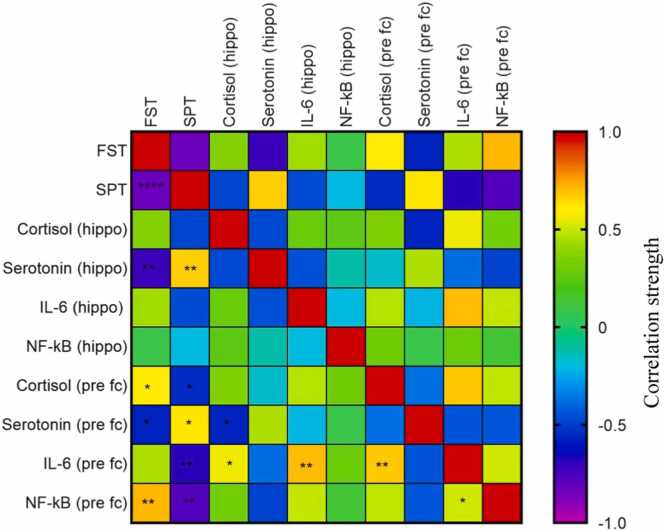
Correlation between ciprofloxacin-induced depression-like behavior (FST and SPT) and the levels of neurotransmitter as well as proinflammatory cytokines.

Fig. 4 depicts the interrelationship between the measured variables. The asterisks indicate a statistically significant relationship. The graph illustrates the strength of the association between neurotransmitters and proinflammatory variables that significantly correlate with depression-like behavior.

### 3.5 Possible mechanisms of ciprofloxacin-induced depression-like behavior and the target action of lactulose in inhibiting depression-like behavior

**Fig. 5:**
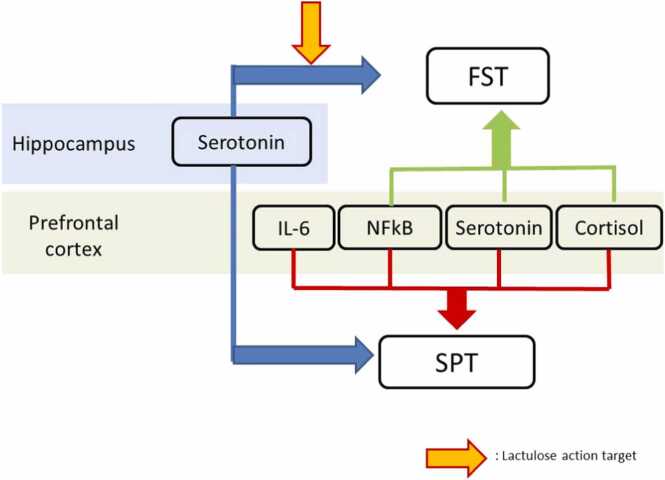
Proposed mechanism of ciprofloxacin-induced depression-like behavior and the site of action of lactulose.

The depression-like behavior induced by ciprofloxacin is significantly influenced by serotonin in the hippocampus; cortisol, serotonin, and NF-κB in the prefrontal cortex, as observed in the FST. However, when viewed from the SPT, the influence of these variables is further complicated by the presence of additional variables affecting IL-6 (Fig. 5). Significant variables automatically also show their influence on depression-like behavior. Following the correlation test on the variables that significantly influence the occurrence of depression, a correlation test was conducted on lactulose in the positive variable as the mechanism of action of ciprofloxacin to induce depression. The results indicated that lactulose inhibits the occurrence of depression-like behavior through the increase of serotonin levels in the hippocampus (Table 1).

**Table 1:** The target of lactulose action in inhibiting depression-like behavior.

**Correlation**	**p**	**R**^**2**^
Serotonin (hippocampus) vs. SPT	0.274	0.527
Serotonin (hippocampus) vs. FST	**0.004**	**0.9923**
Cortisol (prefrontal cortex) vs. SPT	0.812	0.035
Serotonin (prefrontal cortex) vs. SPT	0.223	0.604
NF-κB (prefrontal cortex) vs. SPT	0.743	0. 066
Cortisol (prefrontal cortex) vs. FST	0.299	0.491
Serotonin (prefrontal cortex) vs. FST	0.078	0.851
IL−6 (prefrontal cortex) vs. FST	0.149	0.724
NF-κB (prefrontal cortex) vs. FST	0.153	0.718

## 4 Discussion

Ciprofloxacin, a broad-spectrum antibiotic, presents a paradox within the context of the gut-brain axis mechanism. While effectively treating infections, ciprofloxacin disrupts intestinal microbiota balance, leading to dysbiosis. This subsequently triggers depression via elevated levels of proinflammatory cytokines and alterations in brain neurotransmitter levels [7], [39].

In this study, we employed forced-swim and sucrose preference tests to measure the depression-like behavior in experimental animals. While alternative tests such as open field tests [45], activity cage, rotary rod tests [40], tail suspension test [27], or tail suspension test [41] are viable options, the forced-swim test stands out as the most commonly utilized method. Concurrently, sucrose preference serves as an indicator of anhedonia [42]. Consequently, both behavioral tests are suitable for evaluating depression levels.

The FST assessment using scores demonstrates results encompass immobility, swimming, and climbing, representing active behaviors primarily regulated by distinct neurotransmitter systems. Catecholamine antidepressants can selectively enhance climbing behavior, whereas serotonergic agents have the effect of increasing swimming behavior. Consequently, utilizing the scoring method in FST analysis will prove beneficial in determining whether a novel pharmacological agent predominantly activates one of these neurotransmitter systems.

The present study utilized IL-6 and NF-κB proinflammatory cytokines, alongside serotonin and cortisol as neurotransmitter markers, given their involvement in depression pathology [43], [44]. Diminished serotonin, elevated cortisol, and heightened inflammatory cytokines serve as markers of depression [45], [46], [47].

The administration of antibiotics disrupts the balance of gut microbiota, resulting in the altered production of inflammatory factors that induce depression [48]. These peripheral inflammatory factors can penetrate the blood-brain barrier or activate the Hypothalamus-Pituitary-Adrenal (HPA) axis, elevating glucocorticoid hormone levels. Furthermore, HPA axis activation can reduce neurotrophic factors in the brain, contributing to depression. Proinflammatory cytokine changes have been documented, such as NF-κB activation in the central nervous system [39]. Previous studies have also indicated reduced serotonin levels in the rat hippocampus following treatment with mixed antibiotics, suggesting an association between serotonin and antibiotic-induced depression-like behavior [49].

It is important to note that the methodology employed in this study did not entail using a healthy control for comparison, which is a standard practice in research on depressive behavior. This was because the study's objective was to focus on the mechanism of action of the inducers and treatment target using a correlation test analysis approach to determine it. Consequently, using a healthy control or administering ciprofloxacin on day 0 was not performed. In order to gain insight into the overall pattern of depressive states occurring at various times, it was necessary to observe day increments from days 1–22 to reach definitive conclusions regarding the inducers' mechanism of action and the treatment's action target.

The administration of antidepressants was not included in this study because the depressive behavior observed is thought to be caused by changes in the microbiota in the gut. Restoring the disturbed microbiota balance is a prudent approach to selecting an appropriate treatment. Thus, the administration of antidepressants that act directly on the nerve center is considered an inappropriate comparison. Given the absence of a commercial drug that has unequivocally improved the gut microbiota, which is directly correlated with the onset of depression, the authors sought to identify novel treatments that can improve the gut microbiota and are directly correlated with the alleviation of depressive symptoms. One potential strategy was the use of prebiotics.

In the present study, we tried to employ a novel mechanism for determination. Instead of comparing treatment and control groups, we investigated the mechanism across varying administration durations while maintaining a fixed dose. Our analysis employed multivariate correlation to elucidate the underlying mechanism pathway. This methodology can also be extended to scenarios involving different dosage regimens but uniform administration durations. Conventional methods could provide different mechanism pathways, especially when comparing lower and higher doses with a control group. Lower doses may exhibit less pronounced changes in activity compared to higher doses when compared against a control group. Therefore, using prolonged administration durations with consistent doses or varying doses with uniform administration durations correlated using multivariate correlation tests provides a more nuanced understanding of the mechanism pathway than conventional methodologies. This is because correlation and regression can investigate the causal relationship and strength of association among various parameters [50], [51].

Once the mechanism of action of the inducing substance has been identified and the variables that play a significant role in inducing have been determined, once the mechanism of action of the triggering agent is identified and the variables that play a significant role in triggering are determined, the target action of the treatment can be determined. However, when we examine the method of determining the mechanism generally used (conventional methodologies), which only compares the treatment group with the comparison control, it becomes evident that there is no correlation between the variables. This approach needs to be revised to capture the relationship between variables. Other variables not included in the study may influence the observed effect. This approach is consistent with the principles of mathematical and statistical logic since a disorder might have confounders that influence the possible etiologies.

Patients experiencing depression frequently report constipation, which is attributed to reduced intestinal motility [52]. There is a reciprocal relationship between gut microbiota and constipation, where gut microbiota can influence the occurrence of constipation, and constipation can change gut microbiota [53]. Lactulose, known for its non-absorbability in the small intestine and presence in the large intestine, has been demonstrated to enhance intestinal motility and act as a prebiotic [21]. Low doses of lactulose increase the proliferation of Bifidobacterium and Lactobacillus spp. and decrease the growth of pathogenic clostridia bacteria in the gut [54], [21]. The dosage given in this study has referred to the two effects of lactulose: laxative and prebiotic. In addition, the present study demonstrated a preventive effect of lactulose against ciprofloxacin-induced depression-like behavior, which may be attributed to its role in stimulating bacterial growth and subsequent production of short-chain fatty acids (SCFA) [55]. Among these SCFAs, acetate, propionate, and butyrate have been linked to anti-inflammatory and laxative agents [56], [57], which may explain the ameliorative effect of lactulose on the depression-like behavior observed in the rat model.

Future investigations will explore the impact of short-term intermittent antibiotic administration on gut microbiota and depression-like behavior, providing insights into conditions involving non-compliant patients (a significant factor in microbial resistance) and the precise timeframe for peak depression-like behavior occurrence.

## 5 Conclusions

Our findings indicate that an extended duration of ciprofloxacin induction does not necessarily correlate with heightened depression-like behavior. The results of the SPT indicated that depression-like behavior induced by oral ciprofloxacin was affected by serotonin levels in the hippocampus and serotonin, cortisol, and NF-κB levels in the prefrontal cortex area. Testing using the FST revealed that depression-like behavior was affected by serotonin levels in the hippocampus and serotonin, cortisol, IL-6, and NF-κB levels in the prefrontal cortex area. Finally, our study provides definitive evidence that lactulose prevents ciprofloxacin-induced depression-like behavior by modulating serotonin levels in the hippocampus region.

## Funding

This work was supported by The Education Funding Service Center, the Ministry of Education, Culture, Research and Technology, and the Education Fund Management Institute, Ministry of Finance of the Republic of Indonesia, registration number 202101121157.

## CRediT authorship contribution statement

**Anggadiredja Kusnandar:** Writing – review & editing, Validation, Supervision, Investigation, Data curation. **Sasongko Lucy:** Writing – review & editing, Validation, Supervision, Investigation, Data curation. **Rahman Havizur:** Writing – original draft, Methodology, Funding acquisition, Conceptualization.

## Declaration of Competing Interest

The authors declare that they do not have any competing financial interests or personal relationships that could potentially bias the findings presented in this paper.

## Data Availability

All data supporting the research is presented in this article
